# Clinical characteristics and outcomes of hospitalized COVID-19 patients with diabetes mellitus in East Java, Indonesia: A cross-sectional study

**DOI:** 10.12688/f1000research.111047.1

**Published:** 2022-06-21

**Authors:** Erwin Astha Triyono, Joni Wahyuhadi, Jongky Hendro Prajitno, Hermina Novida, Nenci Siagian, Cupuwatie Cahyani, Arinditia Triasti Putri, Michael Austin Pradipta Lusida, Amal Arifi Hidayat, Karisma Septari Idamusaga, Nastiti Imana Intansari, Jose Asmara, Agrasenfani Hadi, I Ketut Mega Purnayasa Bandem

**Affiliations:** 1 Department of Internal Medicine, Faculty of Medicine, Dr. Soetomo Teaching Hospital, Airlangga University, Surabaya, 60115, Indonesia; 2 Department of Neurosurgery, Faculty of Medicine, Dr. Soetomo Teaching Hospital, Airlangga University, Surabaya, 60115, Indonesia; 3 Department of Cardiology, Faculty of Medicine, Dr. Soetomo Teaching Hospital, Airlangga University, Surabaya, 60115, Indonesia; 4 Indrapura Forefront Hospital, Surabaya, 60175, Indonesia

**Keywords:** ACE2, blood glucose, diabetes mellitus, hyperglycemia, SARS-CoV-2

## Abstract

**Introduction**: Diabetes mellitus has been perceived as the worsening factor for coronavirus disease 2019 (COVID-19), where diabetes mellitus patients with pre-existing inflammatory condition could develop acute respiratory disease syndrome as well as multi-organ dysfunction. Managing diabetes mellitus amidst severe acute respiratory syndrome coronavirus 2 (SARS-CoV-2) infection is also a matter of concern as several antidiabetic therapies could affect the progression of COVID-19. This study aimed to provide the clinical characteristics and outcomes of patients with both COVID-19 and diabetes mellitus receiving blood glucose lowering therapies and COVID-19 symptomatic treatments.

**Methods**: This retrospective study was performed on 260 medical records of patients hospitalized between May 2020 to February 2021 in East Java, Indonesia. Patients were confirmed COVID-19 positive based on the results from real time polymerase chain reaction (RT-PCR) using nasal swab samples collected on hospital admission. Data included were demographic characteristics, COVID-19 symptoms, severity of COVID-19, comorbidities (other than diabetes mellitus), fasting blood glucose (FBG), and 2-hours post-prandial blood glucose (2hPBG), and outcomes.

**Results**: Most of the patients had age range of 41–60 years old (76.1%) with more than a half of the subjects (60%) were obese. Patients with uncontrolled diabetes were distributed evenly among the COVID-19 severities (74.3% in asymptomatic group, 73.6% in mild group, and 74.1% in moderate group). There were reductions in FBG and 2hPBG levels measured before (210.75±81.38 and 271.19±100.7 mg/dL, respectively) and after the treatment (181.03±68.9 and 222.01±86.96 mg/dL, respectively). All patients received multivitamin and symptomatic treatment for COVID-19. Oral antidiabetic drug (57.6%) and insulin (28.8%) were administered to lower the blood glucose level of the patients. As many as 96.9% patients survived, while 3.1% died.

**Conclusion**: COVID-19 could affect the blood glucose level, suggesting the importance of antihyperglycemic therapies among patients with both COVID-19 and diabetes mellitus.

## Introduction

A novel coronavirus severe acute respiratory syndrome coronavirus 2 (SARS-CoV-2) that causes coronavirus disease 2019 (COVID-19) has been responsible to almost 153,000 mortalities in Indonesia as of March 2022 based on Indonesian Government database and has caused many disruptions in the communities. ^1^^,^^2^ SARS-CoV-2 uses angiotensin-converting enzyme 2 (ACE2) as the receptor, in which the enzyme is available in various organs (such as lungs, heart, kidneys, intestines, and so on). ^3^ Diabetes mellitus is a condition responsible for high number of global morbidities, especially due to vascular diseases it induces through chronic inflammation. As any other underlying diseases, diabetes mellitus could contribute to the poor prognosis of COVID-19. ^4^^–^^6^ This is ascribed to the role of inflammation in the pathogenesis of severe COVID-19, where chronic inflammation is a common condition in individuals with diabetes mellitus. ^4^ Common comorbidities found in diabetes mellitus, such as obesity and hypertension, have been evidenced to be responsible for acute respiratory disease syndrome (ARDS) as well as multi-organ dysfunction. ^5^

Understanding clinical characteristics of diabetes mellitus of patients who are infected with SARS-CoV-2 is important to provide proper management. For example, those with poor glycemic control could have worse viral infections, as proven by SARS and influenza H1N1 cases. ^4^ Respiratory distress induced by the viral infection could lead to the apoptosis of pancreatic beta cells that consequently causes insulin insufficiency. ^7^ Therefore, glucose-lowering therapies should be continued or performed during the COVID-19 management. Nonetheless, clinicians should also consider the pro-inflammatory effect of some antidiabetic drugs which could contribute to the progression of severe COVID-19. Thiazolidinediones (TZDs) is one of the antidiabetic therapies that has been found to induce inflammation by elevating ACE2 and angiotensin 1-7 expressions. ^8^ Moreover, in a meta-analysis of 13 trials, increased risk of developing pneumonia was found in TZD group. ^9^ These explanations suggest that there are strong associations between COVID-19 and both diabetes mellitus and its management.

In the case of COVID-19, based on a meta-analysis, the number of patients with diabetes mellitus could reach 8% of the total patients. ^10^ However, the prevalence was dramatically higher (36%) in Italian population, where 34% of which died during the treatment. ^11^ In Indonesia itself, the data from Jakarta province (n=20,481) revealed that the prevalence was only 3.4%, but the mortality rate was higher in diabetes mellitus group (21.28%) than that in non-diabetes mellitus group (2.77%). ^12^ By using a larger data set from the Indonesian COVID-19 Task Force, a study revealed that diabetes mellitus as the second most common comorbidity (33.6%) after hypertension (52.1%). ^13^ Taken altogether, it is still uncertain whether the prevalence of diabetes mellitus among COVID-19 patients, especially in Indonesia, is high. Herein, we reported the data from East Java Province, Indonesia, regarding the clinical characteristics of the COVID-19 and diabetes mellitus patients and the treatment they received during the hospitalization. Moreover, we also reported the outcomes from symptomatic management for COVID-19 in combination with diabetes mellitus therapies which could be recommended for hospitals in developing countries with limited medical and financial resources.

## Methods

### Study design and setting

This study was a retrospective study using the medical records of COVID-19 patients with diabetes mellitus who underwent a hospitalization from May 2020 to February 2021 in Indrapura Forefront Hospital Surabaya, East Java Province – Indonesia (n=260). Patients were confirmed COVID-19 positive by real time polymerase chain reaction (RT-PCR) through nasal swab admission. Diabetes mellitus was confirmed by the officially recorded medical history. The obtained data were collected from electronic medical records including demographic, treatments, laboratory results as well as clinical outcomes. All patients with COVID-19 enrolled in this study were diagnosed and managed according to the national guidelines. This study had received ethical approval from the Ethics Committee of the Faculty of Medicine, Airlangga University, Surabaya, Indonesia (registration number: 37/EC/KEPK/FKUA/2021). Since this study collected the data from the available medical records, the Ethics Committee waived of consent from the patients and this allows by the Indonesian law.

### Study measures

Data used in this study were collected from electronic medical records. Demographic characteristics of the patients included age, sex, and occupation. Clinical characteristics extracted from the medical records were body mass index (BMI), comorbidities, and COVID-19 symptoms. Comorbidities included hypertension, cardiovascular diseases, chronic kidney diseases, and asthma. As for the COVID-19 symptoms, they were cough, fever, rhinorrhea, anosmia, dyspnea, nausea, and headache. Severity of the COVID-19 was classified following the national guideline; asymptomatic, mild, moderate, and severe. Patients showing no COVID-19 symptoms were assigned to asymptomatic groups. Mild COVID-19 was labeled to those who were presenting mild symptoms (such as fever, cough, and nausea) without dyspnea. Patients having manifestation of pneumonia and oxygen saturation ≥93% fell under moderate category. The patients with severe COVID-19 presented with pneumonia accompanied by one of the followings: respiratory rate > 30 times/minute, severe respiratory distress, or oxygen saturation <93%. Fasting blood glucose (FBG) and 2-hours post-prandial blood glucose level (2hPBG) of the patients were measured during the 24 hours of the admission (first measurement) and the last day prior to hospital discharge (second measurement). Changed values of FBG (∆FBG) and 2hPBG (∆2hPBG) were determined by subtracting the value obtained from the first measurement with that from the second measurement. Herein, we also collected the data pertaining to the previous treatment and antihyperglycemic agents received by the patients including their changes during the hospitalization. Length of stay was assigned as the outcome of the treatment, in addition with the COVID-19 positivity. Patients were grouped based on the RT-PCR results on the hospital discharge; patients recovered with COVID-19 negative result and patients discharged with COVID-19 positive results and required additional self-quarantine or referred to another healthcare facility.

### Data analysis

Results of this study were processed on SPSS software version 24 (SPSS Inc., Chicago, IL, USA) (SPSS, RRID:SCR_019096) and expressed as descriptive data. All continuous data were presented in mean ± standard deviation (SD). Meanwhile, categorical data were presented in numbers and percentages.

## Results

Demographic characteristics of the COVID-19 patients with diabetes mellitus (n=260) have been presented in Table 1. The average patients’ age was 51.33 ± 8.85 years old, predominated by those who were 41–60 years old (76.1%) and followed by 61–80 years old group (13.1%) and 21–40 years old group (10.8%). The numbers of female and male patients were almost similar with percentages of 46.2% and 53.8%, respectively. Most of the patients were self-employed (45%), civil servants (19.2%), and housewives (13.8%).

**Table 1.** Demographic characteristics of COVID-19 patients with diabetes mellitus.

Variable	n (%)
Age, mean ± SD (years old)	51.33 ± 8.85
21–40 years old	28 (10.8)
41–60 years old	198 (76.1)
61–80 years old	34 (13.1)
Sex	
Male	140 (53.8)
Female	120 (46.2)
Occupation	
Student	10 (3.84)
Civil servant	50 (19.2)
Teacher	16 (6.2)
Healthcare provider	10 (3.84)
Self-employed	117 (45.0)
Housewife	36 (13.8)
Others	21 (8.07)

As many as 156 (60%) of the total patients were categorized as obese, whereas 55 (21.2%), 36 (13.8%), and 11 (4.2%) others fell into overweight, normal, and underweight categories, respectively ( Table 2). Hypertension was observed as the most common comorbidity (47.7%) recorded upon the hospital admission. There were close numbers of patients between those who were having 1 comorbidity (46.2%) and those who were having 2-3 comorbidities (53.8%). From the highest to the lowest frequency, the COVID-19 symptoms presented included cough (47.7%), fever (23.8%), rhinorrhea (15.4%), dyspnea (13.1%), anosmia (11.5%), nausea (10.0%), and headache (9.6%). As many as 65 (25.0%) patients had COVID-19 symptoms other than those aforementioned ( Table 2).

**Table 2.** Clinical characteristic and blood glucose level of COVID-19 patients with diabetes mellitus (n=260).

Variable	n (%)
Body mass index (BMI)	
Obese	156 (60.0)
Overweight	55 (21.2)
Normal	36 (13.8)
Underweight	11 (4.2)
Comorbidity	
Hypertension	124 (47.7)
Cardiovascular disease	10(3.8)
Chronic kidney disease	1 (0.4)
Asthma	5 (1.9)
Others	14 (5.3)
1 comorbidity	120 (46.2)
2–3 comorbidities	140 (53.8)
COVID-19 Symptoms	
Cough	124 (47.7)
Fever	62 (23.8)
Rhinorrhea	40 (15.4)
Anosmia	30 (11.5)
Dyspnea	34 (13.1)
Nausea	26 (10.0)
Headache	25 (9.6)
Others	65 (25.0)
Severity, n (%) [uncontrolled diabetes, n (%)]	
Asymptomatic	70 (26.9) [52 (74.3)]
Mild	144 (55.4) [106 (73.6)]
Moderate	27 (10.4) [20 (74.1)]
Severe	19 (7.3) [11 (57.9)]
Blood glucose level, mean ± SD (mg/dL)	
First FBG/last FBG	210.75 ± 81.38/181.03 ± 68.9
First 2hPBG/last 2hPBG	271.19 ± 100.7/222.01 ± 86.96
∆FBG	33.14 ± 66.0
∆2hPBG	49.5 ± 89.6

According to the severity, most of the patients suffered mild COVID-19 (55.4%), followed by asymptomatic (26.9%), and moderate (10.4%). There were only 19 (7.3%) patients who were diagnosed with severe COVID-19. Individuals with asymptomatic, mild, and moderate levels of COVID-19 had similar proportion of patients with uncontrolled diabetes (74.3%, 73.6%, and 74.1%, respectively). Meanwhile, the proportion of patients with uncontrolled diabetes was fewer in severe COVID-19 group (57.9%). There was reduction of FBG and 2hPBG levels observed before (210.75 ± 81.38 and 271.19 ± 100.7 mg/dL, respectively) and after the treatment (181.03 ± 68.9 and 222.01 ± 86.96 mg/dL, respectively). Higher mean reduction value was obtained in ∆2hPBG (49.5 ± 89.6 mg/dL) as compared with that in ∆FBG (33.14 ± 66.0 mg/dL).

**Figure 1.**
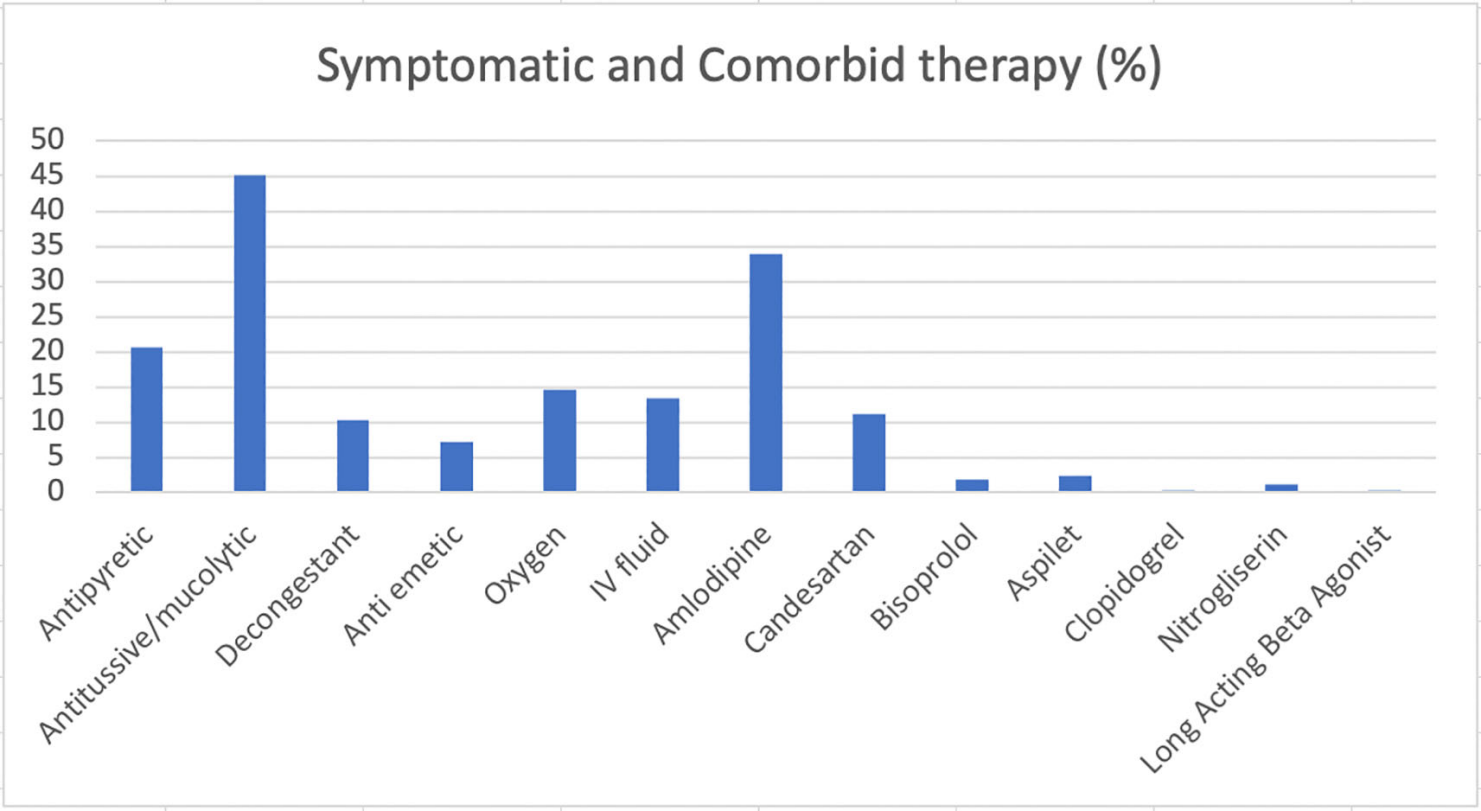
Therapies performed to treat COVID-19 symptoms and comorbidities of the patients during the hospital admission.

**Figure 2.**
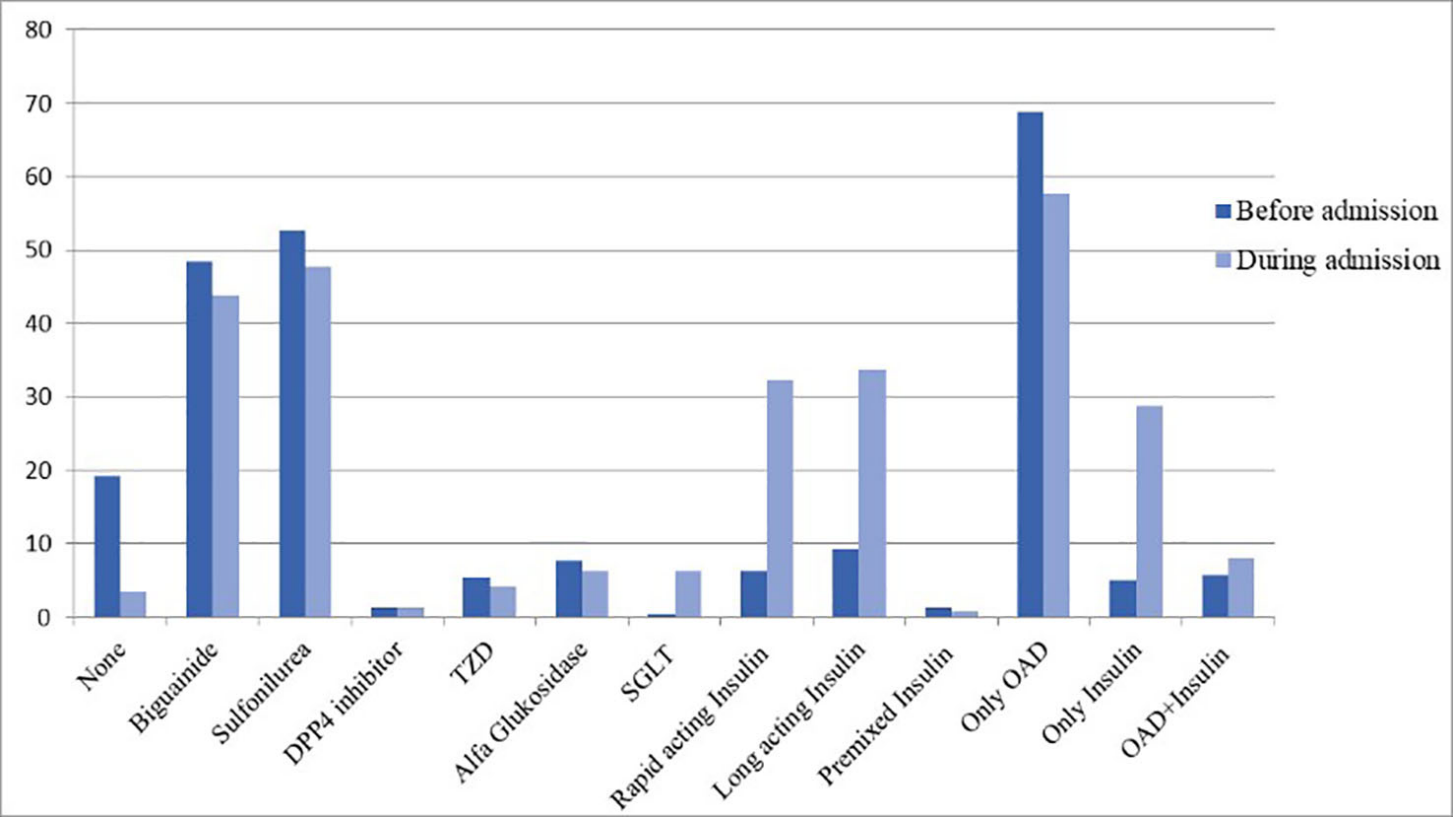
Diabetes mellitus therapies performed on the patients before and during hospital admission. DPP4 = dipeptidyl peptidase-4; OAD = oral antidiabetic drug; SGLT = sodium-glucose linked transporter; TZD = thiazolidinedione.

Therapies performed on the patients during the hospitalization to manage the COVID-19 symptoms as well as comorbidities have been presented ( Figure 1). All patients herein were prescribed with multivitamin. Most of the patients were treated for their COVID-19 symptoms with antitussive/mucolytic agents (47.3%) and antipyretic agents (24.5%), where 31 of the patients (11.9%) received oxygen support. Frequencies of diabetes mellitus treatments received by the patients before and during the hospitalization have been presented ( Figure 2). There were 150 (57.6%) and 75 (28.8%) patients who were given oral antidiabetic drug and insulin, respectively, where 22 (8.4%) others received both therapies. None of the patients included in this study were given antivirals.

Clinical outcomes of the COVID-19 patients with diabetes mellitus have been presented in Table 3. A total of 202 (77.7%) patients were treated in the hospital for more than 10 days. There were 22 (8.5%) patients who underwent the hospital treatment within 10 days. Meanwhile, 36 (13.8%) patients had the total hospital stay shorter than 10 days. As for the outcome, most of the patients were discharged from the hospital with COVID-19 negative results (88.1%). Only 2 (0.8%) patients who were discharged from the hospital and required for self-quarantine following the COVID-19 positive results. There were few individuals referred to the other healthcare facility who then survived (8.1%) and died (3.1%).

**Table 3.** Clinical outcomes of COVID-19 patients with diabetes mellitus (n=260).

Variable	n (%)
Length of stay	
<10 days	36 (13.8)
10 days	22 (8.5)
>10 days	202 (77.7)
Outcomes	
Recovered (negative RT-PCR)	229 (88.1)
Recovered (self-quarantine)	2 (0.8)
Referred & survive	21 (8.1)

## Discussion

Herein, patients presented with cough and fever as the most common symptoms during the onset of COVID-19. ^14^ These results were similar to that of a previous report investigating 904 patients with COVID-19 and diabetes mellitus in China. ^15^ According to multiple reports, combination of COVID-19 and diabetes is fatal because the diseases could complement one another. ^5^ Most patients in this present study were in age group of 41–60 years old (76.1%) with an average of 51.33 ± 8.85 years old. In combination with pre-existing health conditions, such as obesity (60%) and hypertension (47.7%), COVID-19 patients with diabetes mellitus were at higher risk of poor prognosis. ^16^ Obesity could cause poor outcomes in COVID-19 patients with diabetes mellitus because of its association with chronic inflammation. ^4^ Hypertension could downregulate the expression of ACE2, which subsequently increases levels of angiotensin-2 and decreases angiotensin 1-7, leading to the worsening of ARDS. ^17^ Upon the SARS-CoV-2 infection, ACE2 was also found to have decreased, ^18^ becoming an interplay between COVID-19 and hypertension in causing multiple organ failures. ^19^

Despite the fact that individuals with diabetes mellitus could have higher risk to develop severe COVID-19, ^5^ our current findings suggest that only a small percentage of patients reported developing severe COVID-19 (7.3%). Most of the patients had mild (55.4%) and asymptomatic COVID-19 (26.9%). When glycemic control was observed, the majority of patients in asymptomatic, mild, and moderate COVID-19 groups were predominated by individuals with poor blood glucose control (>70%). It is worth mentioning that hyperglycemia could induce the glycation of ACE2, contributing to increased entry of SARS-COV-2 into the host cells. ^20^ Our obtained data suggest that there is no association between diabetes mellitus or hyperglycemic condition with the severity of COVID-19. During the time frame of this study, people have been already aware of COVID-19 and massive testing was carried out. ^21^ We argue that early detection of SARS-CoV-2 infection could prevent the development and the progression of the disease.

Prior to the hospital admission, more than 80% of the patients have received glucose-lowering agents. However, the FBG and 2hPBG were found to be in high levels during the admission. A study found that COVID-19 could cause a dysregulation of lipid metabolism which eventually contributes to insulin resistance. ^22^ Another study reported that as a result of ACE2 downregulation following the SARS-CoV-2 infection, insulin resistance could be developed owing to the exaggeration of angiotensin II. ^23^ During the treatment in the hospital, there was a significant increase of patients receiving sodium-glucose linked transporter (SGLT) inhibitors and insulin therapies. Following the recovery from COVID-19 symptoms, patients showed a reduction of FBG and 2hPBG levels, though they were still far higher than the normal ranges (80–130 mg/dL and <180 mg/dL for FBG and 2hPBG, respectively). ^24^ Here, we could conclude that it is important to control the blood glucose level and maintain diabetes mellitus treatments during the course of COVID-19, as advised by the international panel of diabetes experts. ^4^ Secondly, our data suggests that COVID-19 could influence the level of the blood glucose in diabetic patients.

In this study, there was no specific treatment for COVID-19, only vitamins and symptomatic treatment. None of the patients were given antivirals as there is currently no antiviral specified to treat SARS-CoV-2 infection. The majority of patients (97%) survived after the hospital treatment. As the limitation, with a small number of patients included in this study, it is impossible to draw conclusions that have clinical implications. Within the time frame of the observation, COVID-19 variants have emerged, ^25^ which could contribute to the biased results.

## Conclusions

Most of the patients with both COVID-19 and diabetes mellitus were over 50 years old, and hypertension and obesity were commonly found preexisting conditions in them. Blood glucose level could be increased by the COVID-19, suggesting the importance of blood glucose lowering therapies in diabetic patients with COVID-19. Symptomatic management without antiviral and antibiotic therapy followed by blood glucose lowering therapies contribute to the survivability of the patients.

## Data availability

### Underlying data

Figshare: ‘Clinical characteristics and outcomes of hospitalized COVID-19 patients with diabetes mellitus in East Java, Indonesia: A cross-sectional study.’ DOI: https://doi.org/10.6084/m9.figshare.19388771. ^26^

Data are available under the terms of the Creative Commons Attribution 4.0 International license (CC-BY 4.0).

This project contains the following underlying data:

- Master Data.xls [Table containing the raw data of the study].
- Master Data.sav [Table containing the raw data of the study and the code book].

### Reporting guidelines

Figshare: STROBE checklist for “Clinical characteristics and outcomes of hospitalized COVID-19 patients with diabetes mellitus in East Java, Indonesia: A cross-sectional study” - https://doi.org/10.6084/m9.figshare.19388975.

All data are available under the terms of the Creative Commons Attribution 4.0 International license (CC-BY 4.0).
