# Supplementary material for: Clinical characteristics and outcomes of hospitalized COVID-19 patients with diabetes mellitus in East Java, Indonesia: A cross-sectional study
Source: F1000Res. 2022 Jun 21;11:684. [Version 1] doi: 10.12688/f1000research.111047.1 (PMC9363975; doi:10.12688/f1000research.111047.1)
Supplement: Peer review history

# Peer review: Reviewer response for version 1

**DOI:** 10.5256/f1000research.122720.r144898

**Published:** 2022-08-09

**Authors:** Kuldeep Dhama

**Affiliations:** 1 Division of Pathology, ICAR-Indian Veterinary Research Institute, Bareilly, Uttar Pradesh, India

The study “Clinical characteristics and outcomes of hospitalized COVID-19 patients with diabetes mellitus in East Java, Indonesia: A cross-sectional study” described the clinical characteristics and determined the outcomes of 260 COVID-19 patients with comorbid diabetes mellitus who received the blood glucose lowering therapies. The study used the medical reports between May 2020 and February 2021 in Indonesia. 

 There are some suggestions / recommendations to improve the quality of the article:

1. The diabetes mellitus diagnosis should be provided in more detail. In the article written: "Diabetes mellitus was confirmed by the officially recorded medical history." This criteria used should be explained.
2. "All patients with COVID-19 enrolled in this study were diagnosed and managed according to the national guidelines." Please explain the guidelines. Provide the references here. 
3. It is not clear the outcome groups. It is written: "Patients were grouped based on the RT-PCR results on the hospital discharge; patients recovered with COVID-19 negative result and patients discharged with COVID-19 positive results and required additional self-quarantine or referred to another healthcare facility". Please revise this sentence. 
4. Please provide the reference of how the BMI was classified into obese, overweight, normal, and underweight. 
5. It is not clear the criteria of "uncontrolled diabetes". Please explain in Method and provide the references.
6. Please explain how previous treatment of diabetes was assessed in this study. 
7. Minor: Please define "SARS" in the second paragraph of Introduction. 

**Summary**

 Diabetes mellitus is an important comorbidity with COVID-19, and COVID-19 also affects blood glucose levels, hence antihyperglycemic therapies for lowering blood glucose levels and COVID-19 symptomatic treatments are of importance for treating such patients.

Is the work clearly and accurately presented and does it cite the current literature?

Yes

If applicable, is the statistical analysis and its interpretation appropriate?

Yes

Are all the source data underlying the results available to ensure full reproducibility?

Yes

Is the study design appropriate and is the work technically sound?

Yes

Are the conclusions drawn adequately supported by the results?

Yes

Are sufficient details of methods and analysis provided to allow replication by others?

Yes

Reviewer Expertise:

Infectious diseases, emerging pathogens.

I confirm that I have read this submission and believe that I have an appropriate level of expertise to confirm that it is of an acceptable scientific standard.

# Peer review: Reviewer response for version 1

**DOI:** 10.5256/f1000research.122720.r141584

**Published:** 2022-07-07

**Authors:** Kurnia Fitri Jamil

**Affiliations:** 1 Universitas Syiah Kuala, Banda Aceh, Indonesia

The suggestions that I can give to the manuscript with the title "Clinical characteristics and outcomes of hospitalized COVID-19 patients with diabetes mellitus in East Java, Indonesia: A cross-sectional study", are:

1. There are some grammatical errors in the text. Authors need to re-examine the manuscript.
2. In my opinion, some of the information provided in the Introduction section are more suitable to be included in the Discussion instead. A lengthy introduction compared to the Discussion might give the impression that available studies in the literature on this matter is more than adequate in contributing the information without the need of the current study.
3. I suggest displaying the figures (figures 1 and 2), in a more attractive form.
4. In my opinion, data or comparison groups are needed for users of single oral anti-diabetic drugs only, and users of antidiabetic drugs with insulin, especially in obese patients.
5. It is better if the authors can provide more updates or additional facts that can be obtained from this study compared to what already been published in the literature, and also presents data from others Asian countries, especially Southeast Asia, as comparison data.
6. I would advise you in the future to use a prospective research design.
7. Reference: correct or cite in full reference numbers 14, 17, 26.

Is the work clearly and accurately presented and does it cite the current literature?

Yes

If applicable, is the statistical analysis and its interpretation appropriate?

Yes

Are all the source data underlying the results available to ensure full reproducibility?

Yes

Is the study design appropriate and is the work technically sound?

Yes

Are the conclusions drawn adequately supported by the results?

Yes

Are sufficient details of methods and analysis provided to allow replication by others?

Partly

Reviewer Expertise:

Internist, Tropical Diseases and Infectious Consultant, Professor in Internal Medicine

I confirm that I have read this submission and believe that I have an appropriate level of expertise to confirm that it is of an acceptable scientific standard.
